# *In situ* synchrotron X-ray scission of polytetrafluoroethylene chains and elucidation of dry etching

**DOI:** 10.1016/j.heliyon.2023.e15794

**Published:** 2023-04-29

**Authors:** Kaito Fujitani, Kento Takenaka, Koji Takahara, Hirosuke Sumida, Akinobu Yamaguchi, Yuichi Utsumi, Satoru Suzuki

**Affiliations:** a Laboratory of Advanced Science and Technology for Industry, University of Hyogo, 3-1-2 Kouto, Kamigori, Ako, Hyogo 678-1205, Japan; b Graduate School of Science, University of Hyogo, 3-2-1 Koto, Kamigori, Ako, Hyogo 678-1297, Japan; c Mazda Motor Corp., 3-1, Shinchi, Fuchu, Aki, Hiroshima 730-8670, Japan

**Keywords:** Polytetrafluoroethylene, PTFE, HAXPES, Synchrotron radiation

## Abstract

We investigated the mechanism of polytetrafluoroethylene (PTFE) chain scission through *in situ* hard X-ray photoelectron spectroscopy at room temperature, 200 °C, and 230 °C. The *C*–C bonds in the main chain and *C*–F bonds in the side chains were broken, and F desorption from the PTFE surface was observed at room temperature. The formation of CF_3_ was also observed from the recombination of broken *C*–C bonds in the main chain and detached F, which were not induced by soft X-rays. In contrast, when the PTFE substrate was irradiated with hard X-rays at 200 °C, the CF_3_ intensity initially produced by recombination reactions decreased with irradiation time, and the photoelectron spectrum retained the original PTFE spectrum. Under these conditions, the F1s/C1s intensity ratio did not change with the irradiation time; hence, the fragment containing only CF_2_, the chemical composition of the original PTFE, was desorbed. When the substrate temperature was 230 °C, the CF_3_ intensity increased in relation to that at 200 °C. This result indicated that the formation of CF_3_ via recombination reactions of broken molecular chains is enhanced by thermal assistance. These phenomena were considered to be based on the balance between recombination and desorption by photochemical and pyrochemical reactions. These results will lead to a better understanding of the use of X-ray-irradiated fluorine resins and PTFE in potential space-based environments. This study will also promote the improvement of PTFE microfabrication methods and thin-film formation using synchrotron radiation.

## Highlights

• PTFE chain hard X-ray scission observed *in situ* via hard XPS.
• Hard X-rays induced main-chain *C*–C cleavage and CF_3_ via recombination reactions.
• *In situ* observation of 200 °C PTFE etching, maintaining chemical composition.
• PTFE etching via recombination–desorption balance upon photo/pyrochemical reactions.

## 1 Introduction

Lab-on-a-chip (LOC) and micro-total-analysis systems (μ-TAS) have been applied in various fields, such as clinical diagnosis, environmental analysis, and chemical synthesis [1]. These systems can be used in chemical synthesis and analysis by connecting unit chemical operations such as mixing, filtration, and heating. Fluorine resins, such as polytetrafluoroethylene (PTFE), are one of the most suitable materials for fabricating LOC systems and μTAS [2,3]. These materials exhibit heat, chemical, and abrasion resistance as well as possess good electrical properties. Especially, composed of *C*–C bonds in the main chain and *C*–F bonds in the side chains, PTFE has a high melting point of 327 °C and resistance to most chemicals, including strong acids such as hydrofluoric acid and aqua regia [3,4]. Therefore, PTFE has applications in aerospace, electrical engineering, medical chemistry, and biology. However, the formation and high-precision microfabrication of PTFE thin films with high aspect ratios are essential for their application in these devices.

Generally, machining methods with and without lasers are used for PTFE microfabrication [5,6]. The former process can remove irradiated areas at high rates; however, the irradiation efficiency per laser pulse is low because of the low focal depth. In the latter case, the low elastic modulus of PTFE impedes its high-precision fabrication. To solve these problems, microfabrication via synchrotron X-ray lithography has been suggested [7], which requires energies of several tens of electronvolts to tens of kiloelectronvolts and sample heating below the melting point to etch patterns in the irradiated area. A 77-μm square hole with an aspect ratio of 50 was formed in this way on a 5-mm-thick PTFE sheet at 200 °C [8]. In addition, synchrotron radiation was used to form a thin film on a Si substrate with the chemical composition identical to that of pristine PTFE at 200 °C [9].

Clarifying the dynamics of the PTFE surface under X-ray irradiation is necessary to optimise the conditions for realising high-precision microfabrication of PTFE under synchrotron radiation. Previous studies mainly investigated the effects of synchrotron radiation on the surface of fluorine resins in a soft X-ray region [[10], [11], [12], [13], [14], [15]]. Haruyama et al. reported the effects of the irradiation dose and heating temperature on the PTFE surface irradiated with soft X-rays through X-ray photoelectron spectroscopy (XPS) [10,11]. Specifically, PTFE samples were irradiated with energies of 0.05–1.0 keV, but had to be exposed to air for transfer to the XPS chamber. In this study, it was found that F was desorbed in vacuum and carbonised by irradiation with soft X-rays [10]. Recently, the process of carbonisation by desorbing the component of the side chain revealed the formation of an amorphous carbon layer [12] and carbon double bonds [13], by measuring polyvinylidene difluoride (PVDF) with X-ray absorption near edge structure (NEXAFS) while continuously irradiating with soft X-rays. In addition, it was discovered that the formation of CF_3_ via recombination reactions of broken molecular chains was promoted when the PTFE substrate was irradiated with soft X-rays at 200 °C [11]. However, it is necessary to consider the effects of hard X-rays because microfabrication via synchrotron radiation can be performed over a wide energy range, from soft to hard X-rays. In addition, *in situ* observation must be conducted to understand the effects of synchrotron radiation because exposure to air may cause oxygen binding [10].

Furthermore, Kato et al. used a quadrupole mass spectrometer to observe fragments produced *in situ* at synchrotron radiation energies widely ranging over 0.4–5.5 keV [16]. Because the main emitted gas was CF_3_, regardless of the substrate temperature (<200 °C), they concluded that the PTFE molecular chain was broken by the X-rays and desorbed as saturated fluorocarbons (CF_3_-CnF_2_m-CF_3_). However, the chemical composition of the irradiated area was not directly analysed; therefore, the electronic state and chemical composition of PTFE surfaces irradiated with synchrotron radiation are not well understood. To understand the effects of irradiation at different energies, it is necessary to analyse surfaces using synchrotron radiation focused on high energies.

Here, the PTFE chemical composition was analysed *in situ* through near-ambient-pressure hard X-ray photoelectron spectroscopy (NAP-HAXPES) to characterise the PTFE surface upon hard X-ray irradiation. The charging effect of PTFE, which is a limiting factor of HAXPES, was eliminated by introducing nitrogen gas and an efficient differential pumping system. *In situ* photoelectron spectra were acquired to identify the effects of synchrotron radiation. The chemical composition and intensity ratios of PTFE were also characterised by examining the temperature dependence under hard X-rays. The effects of hard X-ray irradiation on the PTFE surface and the mechanism of synchrotron dry etching were analysed.

## 2 Materials and methods

**Fig. 1:**
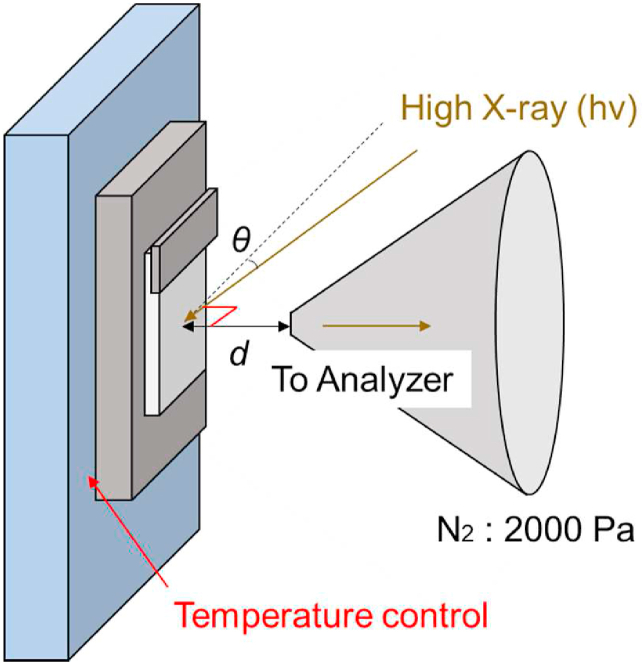
Experimental arrangement for hard X-ray photoelectron spectroscopy of polytetrafluoroethylene.

HAXPES was performed in the BL 24XU undulator at Spring-8 using a HiPP-2 electron energy analyser (Scienta Omicron, Sweden). The X-ray energy was set at 8 keV using a Si channel-cut monochromator. Fig. 1 shows the experimental setup used in the analysis chamber. High-energy X-rays from the beamline were introduced into the analysis chamber through a 200-μm-thick Be filter and focused on the sample surface. The X-ray flux at the sample surface was approximately 2.5 × 10^10^ photons/s in a 30 × 30-μm^2^ beam spot and a 4° angle of incidence *θ*. In addition, the angle between the incident X-ray and the analyser is 90°. The 0.3-mm-diameter aperture cone φ allowed photoelectrons to reach the analyser while efficiently achieving a differential exhaust. Using the Hipp-2 electron energy analyser, gas pressures up to 5000 Pa could be used.

Because PTFE is an insulator material, the spectrum could not be observed owing to the severe charge-up when measured in a vacuum. To eliminate the charging effect, techniques to form a conductive film on the sample surface [17] and irradiate the sample surface with electron beams using a neutralising gun [18] are commonly used. However, the former method inhibits the desorption of small molecules induced by X-ray irradiation, whereas the latter method causes damage to PTFE.

To solve these problems, we considered the use of environmental change compensation [19]. Using this method, which eliminates the charging effect by introducing an inert gas such as nitrogen or argon, it is possible to simultaneously achieve desorption of fragments induced by X-ray irradiation and charging compensation. In our usual experimental setup, the distance *d* from the sample surface to the cone was 0.3 mm [19]. However, the introduction of gas at this distance did not sufficiently eliminate the charge-up and adhesion of desorbed PTFE to the cone. Recently, we found that the effect of environmental charge compensation was more pronounced for a larger length *d* from the tip of an aperture cone to the PTFE substrate, allowing charge-free measurements at lower pressures [20]. In this study, charge-up was eliminated by moving the distance *d* to 1.8 mm and introducing nitrogen at 2000 Pa. In addition, increasing d prevented the irradiation-induced PTFE desorption from adhering to the cone. The pressure in the photoelectron analyser chamber was 1.0 × 10^−5^ Pa when N_2_ gas was introduced at 2000 Pa. The binding energy was adjusted by measuring the Au4f_7/2_ at 84.0 eV. HAXPES has a total energy resolution of approximately 0.3 eV.

Commercial 500-μm-thick PTFE [3,4] sheets (UNIVERSAIL Co., Ltd., 00I-251-02) were mounted in a stainless-steel holder for heating. The spectra were acquired continuously at room temperature to analyse the effects of hard X-rays on the PTFE surfaces over time. The measurement time for F1s was 150 s and that for C1s was 300 s to observe the change in the photoelectron spectrum over time. Narrow scanning analyses of C1s and F1s were successively performed in that order. Spectra were acquired at room temperature, 200 °C, and 230 °C to investigate the effects on the PTFE surface during hard X-ray irradiation.

## 3 Results and discussion

**Fig. 2:**
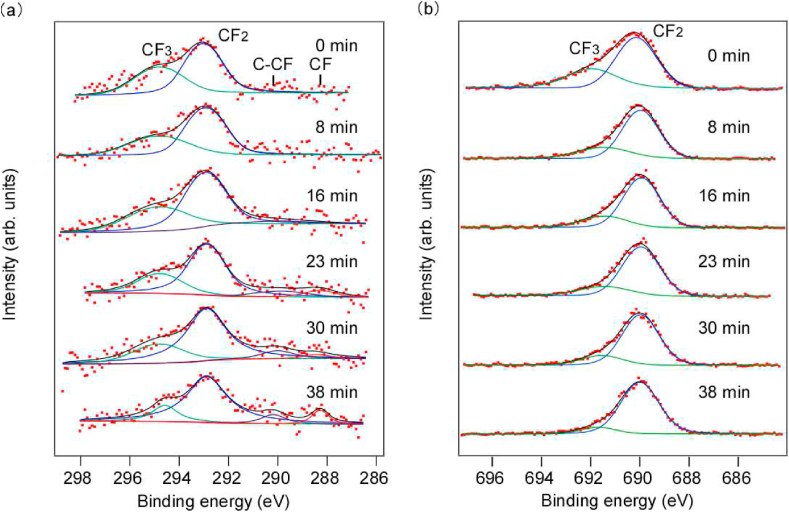
Narrow scanning analysis of (a) C1s and (b) F1s during hard X-ray irradiation at room temperature.

Fig. 2 shows the room-temperature C1s and F1s HAXPES profiles over time. In addition, the relative intensities of C1s at room temperature are summarized in Table 1. Curve fitting using the Voigt function was performed for each spectrum [21]. The weak signal intensity in the C1s spectra was attributed to the introduction of N_2_ gas used for charge elimination, which exponentially decreased the photoelectron intensity with *d*. The 8-keV excitation cross-section of the lighter element C1s was approximately 18% of that for F, which was lower in intensity [22]. In the first C1s spectrum in Fig. 2(a), peaks were observed at approximately 294.8 eV and 292.9 eV binding energies, which were attributed to CF_3_ and CF_2_, respectively [[23], [24], [25], [26]]. At this time, the *C*–C bonds in the main chain were hardly observed. In addition, after continuous irradiation with hard X-rays, the peaks shifted to 290.3 eV and 288.4 eV, respectively, and were attributed to CF and *C*-CF, respectively [[23], [24], [25], [26]]. These results indicate that the *C*–C main chain and the *C*–F side chains were broken on the PTFE surface by hard X-rays, and that CF_3_ was formed by the recombination of the broken molecular chains. Interestingly, the result that the *C*–C peak in the main chain does not appear and the CF_3_ peak appears differs significantly from the XPS spectra of soft X-ray-irradiated PTFE surfaces. Previous reports on room-temperature XPS after soft X-ray (50–1000 eV) irradiation indicated F desorption, and only a carbon-rich *C*–C peak was observed [10]. In addition, CF3, CF, *C*-CF, and CF_2_ peaks were hardly appeared [10]. In contrast, when the 8-keV X-rays were used, the *C*–C peak did not appear, and the CF_3_, CF, and *C*-CF peaks appeared, indicating that the molecular chain scission and recombination reactions differed depending on the X-ray energy. This is considered to be caused by photochemical and pyrochemical reactions due to the continuous irradiation of hard X-rays. Hence, the hard X-rays probably broke the PTFE main chain because the higher energy was more likely to break the *C*–C bonds, with more recombination with F detached by the side-chain scissions.

**Table 1:** Relative area intensity of each component in the C1s, F1s/C1s relative intensity, and CF_3_/CF_2_ relative intensity during hard X-ray irradiation at room temperature.

Irradiation time (min)	CF3 (%)	CF2 (%)	CF (%)	*C*-CF (%)	F1s/C1srelative intensity	CF_3_/CF_2_ relative intensity
0	33.9	66.1	–	–	11.4	0.51
8	29.6	70.4	–	–	12.3	0.42
16	27.6	72.4	–	–	9.4	0.38
23	22.0	67.8	6.0	4.3	9.5	0.32
30	19.7	71.4	6.3	2.6	6.3	0.28
38	12.8	75.4	5.9	5.9	6.1	0.17

Fig. 2(b) shows a narrow scanning analysis of the F1s spectra measured continuously during hard X-ray irradiation. With curve fitting, peaks were observed at binding energies of approximately 691.7 eV and 690.2 eV. The latter peak was attributed to CF_2_, which was present in the main chain of PTFE [14]. However, the peak at 691.7 eV has not been previously reported. When the CF_3_ peak was observed in the C1s spectra [Fig. 2(a)], the 691.7 eV peak was also observed in the F1s spectra [Fig. 2(b)]. Furthermore, the chemical composition ratios of the CF_3_ and CF_2_ peaks in the C1s spectra were 33.9% and 66.1%, respectively. Converting these chemical composition ratios to the ratios of F, CF_3_ and CF_2_ peaks were 43.5% and 56.5%, respectively. On the other hand, in Fig. 2(b), the composition ratios of 691.7 eV and 690.2 eV in the F1s spectra were 46.5% and 53.5%, respectively. The ratio converting chemical composition ratios of CF_3_ and CF_2_ in C1s spectra to the ratio of F was similar to the ratios of 691.7 eV and 690.2 eV in the F1s spectra. Hence, the peaks at 691.7 eV and 690.2 eV appeared to be from CF_3_ and CF_2_, respectively. The CF_3_ peak at 691.7 eV was not observed previously, possibly because the chemical composition ratio of CF_3_ was smaller than that of CF_2_ and overlapped with the CF_2_ peak [25]. Furthermore, because previous reports used *ex situ* analysis [10,11], it is possible that molecular chain scissions or recombination occurred during exposure to air. Hence, the *in situ* measurements here may have enabled the observations.

It was previously reported that F1s peaks appeared at 687.3 eV and 685.3 eV in addition to the CF_2_ peak upon irradiation with soft X-rays [10]. These peaks were attributed to CF and *C*-CF, respectively [10] and were not observed here. In a previous study, PTFE irradiated with soft X-rays was exposed to the atmosphere before XPS measurements. Hence, these sample surfaces were likely to be bound to atmospheric oxygen.

**Fig. 3:**
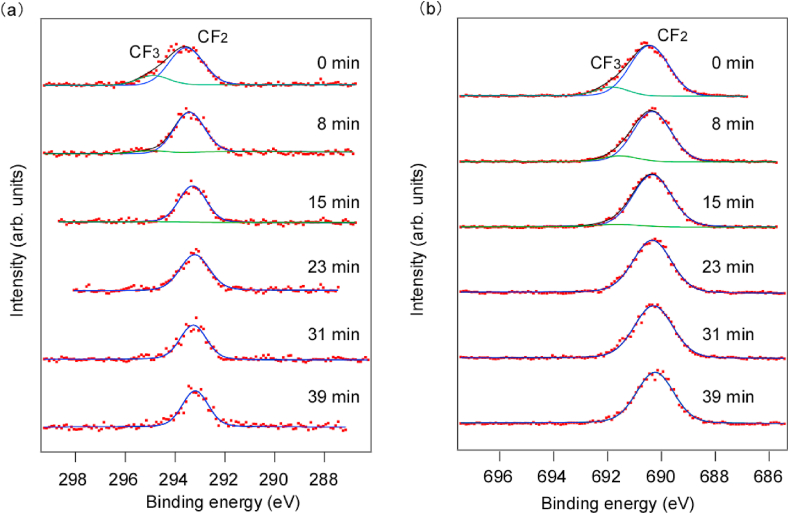
Narrow scanning analysis of (a) C1s and (b) F1s during hard X-ray irradiation at 200 °C.

Fig. 3 shows C1s and F1s photoelectron spectra over time when irradiated with high-energy synchrotron radiation at 200 °C. In addition, the relative intensities of C1s at 200 °C are summarized in Table 2. In the first C1s spectrum in Fig. 3(a), peaks were similar to those acquired at room temperature [Fig. 2(a)], with CF_3_ and CF_2_ peaks at 294.9 eV and 293.2 eV, respectively [[23], [24], [25], [26]]. However, unlike the spectrum at room temperature, the CF_3_ peak decreased over time and only the CF_2_ peak remained. In the previous research, when the substrate temperature was raised 200 °C and irradiated with soft X-rays, CF_3_ *C*–F and *C*-CF peaks in addition to the CF_2_ peak in the main chain of PTFE appeared with soft X-ray irradiation [11]. This result is attributed to the breaking of *C*–C bonds and recombination by pyrochemical reactions. In contrast, when the 8-keV X-rays were used here at 200 °C, the CF_3_, CF, and *C*-CF peaks did not appear, only the CF_2_ peak was observed. This result indicates that the formation of CF_3_ by recombination hardly occurred on the PTFE surface when irradiated with hard X-rays. Hence, it is considered that fragments broken by hard X-rays are detached before recombination by photochemical and pyrochemical reactions. The F1s spectra in Fig. 3(b) shows CF_3_ and CF_2_ peaks at 691.7 eV and 690.3 eV, respectively, which were consistent with the C1s spectra. In previous research, the F1s spectra of PTFE surfaces irradiated with soft X-rays during heating have not been reported; therefore, no comparison can be made. However, CF and *C*-CF peaks observed on the PTFE surfaces irradiated with soft X-rays at room temperature were not observed.

**Table 2:** Relative area intensity of each component in the C1s, F1s/C1s relative intensity, and CF_3_/CF_2_ relative intensity during hard X-ray irradiation at 200 °C.

Irradiation time (min)	CF3 (%)	CF2 (%)	CF (%)	*C*-CF (%)	F1s/C1srelative intensity	CF_3_/CF_2_ relative intensity
0	13.6	86.4	–	–	9.3	0.24
8	7.4	92.6	–	–	7.6	0.08
15	0.3	99.7	–	–	9.0	0.01
23	–	100.0	–	–	9.6	0
31	–	100.0	–	–	9.1	0
39	–	100.0	–	–	9.9	0

**Fig. 4:**
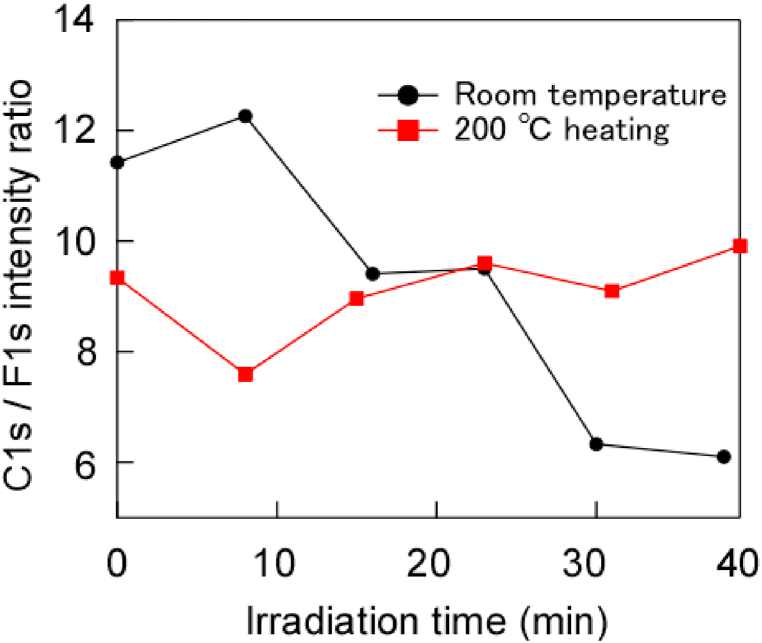
F1s/C1s intensity ratio for polytetrafluoroethylene at room temperature and 200 °C.

Fig. 4 shows the F1s/C1s intensity ratio when irradiated with hard X-rays at room temperature and at 200 °C. The ratio decreased with time when irradiated at room temperature; thus, the desorption of F occurred during carbonisation. However, the *C*–C peak was not observed in Fig. 2, and because the *C*–F, *C*-CF, and CF_3_ peaks appeared, recombination with broken *C*–C and F on the surface must have occurred simultaneously. In contrast, the F1s/C1s ratio during the irradiation at 200 °C did not significantly decrease relative to that at room temperature (Fig. 4). In other words, they were detached as fragments while maintaining the F1s to C1s intensity ratio of the original PTFE. In addition, by integrating the results in Fig. 3(a), it was inferred that PTFE was desorbed (i.e. etched) while maintaining its composition.

Notably, the fragments are desorbed by photochemical and pyrochemical reactions; however, generally, the smaller the average degree of polymerisation, the smaller the melting point of the polymer [27]. Hence, fragments that desorbed at room temperature during hard X-ray irradiation were saturated fluorocarbons (CF_3_- C_n_F_2n_ –CF_3_) of low molecular weight. Whereas, at 200 °C, they desorbed as larger-molecular-weight fluorocarbons (polymers with larger n) than those desorbed at room temperature. In addition, the etching depth was measured using a laser microscope (Keyence Co. Ltd., VK-8510). The sample irradiated with hard X-rays at 200 °C was etched to a maximum depth of approximately 70 μm from the PTFE surface at an etching rate of 1.8 μm/min.

**Fig. 5:**
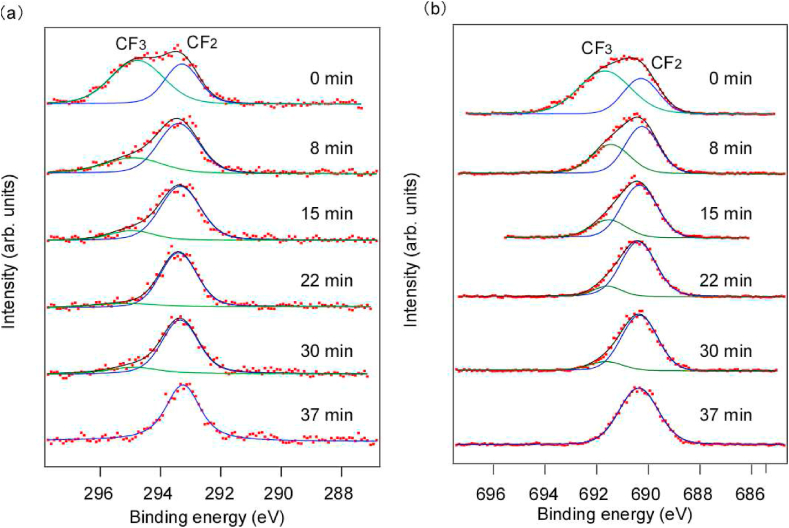
Narrow scanning analysis of (a) C1s and (b) F1s during hard X-ray irradiation at 230 °C.

**Fig. 6:**
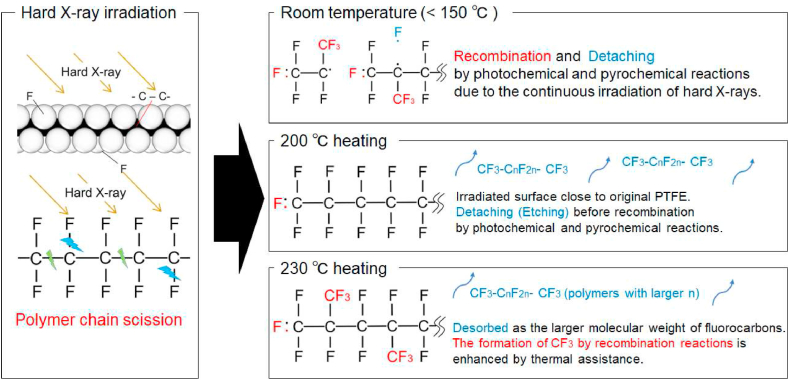
The schematic diagrams of the PTFE surface during hard X-ray irradiation at arbitrary temperatures.

Fig. 5 shows C1s and F1s photoelectron spectra over time when irradiated with high-energy synchrotron radiation at 230 °C. In addition, the relative intensities of C1s at 230 °C are summarized in Table 3. In the C1s spectra in Fig. 5(a), peaks were also observed at 294.9 eV and 293.2 eV peaks, corresponding to CF_3_ and CF_2_, respectively [[23], [24], [25], [26]]. Only the CF_2_ peak was observed later. The secular change was similar to those at 200 °C, but the CF_3_ peak was larger than that at 200 °C. This result indicates that the formation of CF_3_ by recombination owing to the pyrochemical reaction increases with increasing PTFE surface temperature. In a previous study, when the substrate temperature was raised above 200 °C and irradiated with soft X-rays, CF_3_ and *C*–F peaks in addition to the CF_2_ peak in the main chain of PTFE appeared with soft X-ray irradiation [11]. It has also been reported that the formation of CF_3_ by recombination increases with increasing PTFE surface temperature [11]. Integrating previous research, the formation of CF3 by recombination due to pyrochemical reactions increases with increasing surface temperature, regardless of X-ray energy. These results are shown in Fig. 6.

**Table 3:** Relative area intensity of each component in the C1s, F1s/C1s relative intensity, and CF_3_/CF_2_ relative intensity during hard X-ray irradiation at 230 °C.

Irradiation time (min)	CF3 (%)	CF2 (%)	CF (%)	*C*-CF (%)	F1s/C1srelative intensity	CF_3_/CF_2_ relative intensity
0	61.8	38.2	–	–	8.7	1.62
8	30.9	69.1	–	–	8.7	0.45
15	17.1	82.9	–	–	10.9	0.20
22	11.3	88.7	–	–	10.0	0.13
30	16.8	83.2	–	–	9.0	0.20
37	–	100.0	–	–	10.6	0

**Fig. 7:**
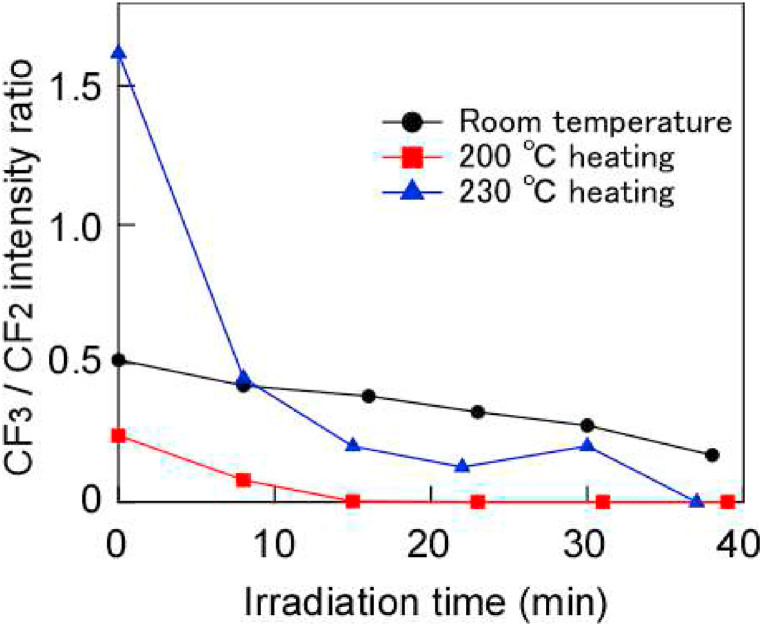
CF_3_/CF_2_ intensity ratio vs. hard X-ray exposure at various temperatures.

Fig. 7 shows the CF_3_/CF_2_ ratio for C1s when irradiated with hard X-rays at various temperatures. This result shows that the ratio decreased over time at all temperatures. It is considered that a new PTFE surface was observed by progressive desorption because of the increase in surface temperature caused by continuous irradiation. In addition, comparing the CF_3_/CF_2_ ratio for C1s at 200 °C–230 °C in Fig. 7, the ratio at 230 °C is larger than that at 200 °C. As mentioned earlier, this is considered to be due to pyrochemical reactions. On the other hand, comparing the CF_3_/CF_2_ ratio for C1s at room temperature to 200 °C in Fig. 7, the ratio at room temperature was larger than that at 200 °C. This result is attributed to the fact that CF_3_ is produced by recombination owing to photochemical reactions. On the other hand, at a CF_3_/CF_2_ ratio of 200 °C, it is considered that the formation of CF_3_ on the surface by photochemical and pyrochemical reactions is balanced by desorption due to these reactions, so CF_3_ was hardly observed. As the temperature increased, the samples were etched as polymeric fragments (CF_3_-C_n_F_2n_-CF_3_), where n increased, and new surfaces appeared.

X-ray lithography for fluorine resins induced by synchrotron radiation causes pattern distortion, collapse, and a decline in accuracy because of the thermal expansion difference between fluorine resins and X-ray masks made of Ni [3,8,28]. In other words, the surface temperature during X-ray irradiation should be as low as possible for high-precision microfabrication. In this study, we found that PTFE was desorbed (i.e. etched) while maintaining its composition when irradiated with high-energy synchrotron radiation at 200 °C. (When measured below 200 °C, C1s spectra were similar to those at room temperature.). In other words, although higher temperatures increased etching rates, a theoretically infinitely high aspect ratio was possible and optimal with X-ray irradiation at 200 °C.

## 4 Conclusions

Changes in the chemical composition of PTFE and its components induced by high-energy X-rays were analysed through *in situ* HAXPES. When hard X-rays were continuously applied at room temperature, scission of the main PTFE chain and side chains was induced, and recombination reactions occurred over time. Hence, CF_3_, CF, and *C*-CF photoelectron peaks are observed on the PTFE surface. At high X-ray energies, *C*–C scissions of the main chain of PTFE were more progressive, and there was more recombination of F that had been desorbed by the side-chain scissions. Upon hard X-ray irradiation at 200 °C, the CF_3_ peak decreased with time, leaving only the CF_2_ and original PTFE peaks. This indicates that the broken PTFE detached as saturated high-molecular-weight fluorocarbons. When the substrate temperature was 230 °C, the CF_3_ peak was larger than that at 200 °C because of pyrochemical effects.

This study will promote the development of methods for high-precision thin-film deposition and microfabrication via synchrotron radiation irradiation. Thus, the scope of application of PTFE thin films in micro-total-analysis, LOC system development, biochemistry, and medicine can be expanded. Furthermore, because of these high-energy X-ray irradiation results, the abrasion resistance, heat resistance, and electrical properties of PTFE can be exploited for its application in space-based materials.

## Author contribution statement

Kaito Fujitani: Conceived and designed the experiments; Performed the experiments; Analysed and interpreted the data; Contributed reagents, materials, analysis tools or data; Wrote the paper.

Kento Takenaka, Koji Takahara: Performed the experiments.

Hirosuke Sumida, Akinobu Yamaguchi, Yuichi Utsumi: Contributed reagents, materials, analysis tools or data.

Satoru Suzuki: Conceived and designed the experiments; Performed the experiments; Analysed and interpreted the data; Contributed reagents, materials, analysis tools or data.

## Data availability statement

Data included in article/supp. Material/referenced in article.

## Additional information

No additional information is available for this paper.

## Declaration of competing interest

The authors declare that they have no known competing financial interests or personal relationships that could have appeared to influence the work reported in this paper
